# Managing persistent physical symptoms when being social and active is the norm: a qualitative study among young people in Denmark

**DOI:** 10.1186/s12889-023-16910-2

**Published:** 2023-10-07

**Authors:** Julie Høgsgaard Andersen, Mette Bech Risør, Lisbeth Frostholm, Mette Trøllund Rask, Marianne Rosendal, Charlotte Ulrikka Rask

**Affiliations:** 1 Research Clinic for Functional Disorders and Psychosomatics, Aarhus University Hospital, Aarhus, Denmark; 2 Department of Child and Adolescent Psychiatry, Aarhus University Hospital, Psychiatry, Aarhus, Denmark; 3 Research Clinic for Functional Disorders and Psychosomatics, Department of Child and Adolescent Psychiatry, Psychiatry, Aarhus University Hospital, Palle Juul-Jensens Boulevard 99, Aarhus N, 8200 Denmark; 4 Department of Public Health, The Research Unit for General Practice, University of Copenhagen, Copenhagen, Denmark; 5 General Practice Research Unit, Department of Community Medicine, UiT The Arctic University of Norway, Tromsoe, Norway; 6 Department of Clinical Medicine, Aarhus University, Aarhus, Denmark; 7 The Research Unit for General Practice, Aarhus, Denmark

**Keywords:** Persistent physical symptoms, Young people, Functional disorders

## Abstract

**Background:**

An increasing number of young people in Western countries report persistent physical symptoms (PPS). PPS may disturb everyday activities and they may have negative consequences for later adult mental and physical health. Still little is known about how young people handle PPS in their everyday lives. This study examines how young people with PPS attempt to manage their symptoms while staying engaged in their daily activities and what is at stake in these attempts.

**Methods:**

This qualitative study involved semi-structured interviews with 11 young people with PPS. Photo-elicitation was used to capture the participants’ experiences as they occurred in their everyday lives. The data material was analysed using a thematic analysis approach, as well as theory on subjectivity and social acceleration.

**Results:**

The participants employed alleviating measures and tried to find patterns between their activities and the severity of their symptoms in order to adjust their activity level. Decisions not to participate in social activities were accompanied by feelings of missing out. The participants’ attempts at adjusting their activity level was challenged by norms of being social and active, and they experienced difficulty prioritizing their activities and explaining their symptoms to others.

**Conclusion:**

PPS shaped the participants’ sense of how to act towards their bodies and social relationships in interaction with societal norms. The participants’ subject formation and symptom experiences should thus be seen as a biosocial process.

## Background

An increasing number of young people in Western countries report unspecific physical symptoms and poor self-rated health [1–4]. In Denmark, a national survey showed a marked increase in 16-24-year-olds who had experienced either bothersome pain, difficulty sleeping, or feelings of being sad, depressed, or unhappy: In 2010, 30% of males and 46.1% of females in this age group experienced these symptoms, in comparison, in 2021, the numbers had increased to 46.4% for males and 61.1% for females [4]. Persistent physical symptoms (PPS) is a newly introduced term for complaints that are not verifiable by clinical or para-clinical findings [5]. PPS are often tied to emotional problems such as anxiety and depression, and they may disturb a person’s participation in everyday activities and may lead to an increased use of healthcare services and medication [6, 7]. Furthermore, severe PPS and low self-reported health early in life may have negative consequences for adult mental and physical health, including the development of functional disorders [8, 9].

Patients with PPS constitute a large proportion of patient contacts in general practice [10–12]. At present, there is no formalized treatment of PPS in general practice in Denmark, and general practitioners (GPs) tend to find the management of the symptoms in this patient population burdensome and time consuming [12].

The web-based self-help programme ‘*My Symptoms*’ targeting adults with PPS has been developed recently. This programme is based on the principles of cognitive behavioural therapy (CBT) and graded exercise therapy known to have positive effects on functional disorders [13]. The present study is part of the research project *Yo-eASY (Young ehealth and GP Assisted programme for unspecific Symptoms)* aiming to redesign the My Symptoms programme in order to make it suitable for young people aged 15–25 years. The study provided a basis for the redesign by exploring how young people with PPS attempt to manage their symptoms as well as what is at stake for them while trying to stay engaged in their daily activities.

### Qualitative research on young people with persistent physical symptoms

Qualitative research on young people with PPS has contributed to our knowledge on how young people understand and explain PPS [14–17], on the consequences of having PPS in their daily lives, including disruptions in educational paths, after-school activities, and social relations [15, 16, 18], and on their engagement in therapeutic processes [14, 19, 20]. Most of the existing studies have focused on young people in hospital-based treatment, but Ostbye [16] and Kvamme [15] have studied young people’s efforts at making sense of and coping with PPS in an everyday setting. They showed how young people associate PPS with adverse life events, personal and social problems, and how they try to carve out new developmental paths for themselves and become accountable young persons. Still, knowledge on how young people experience and attempt to manage PPS in their everyday lives is sparse. Research has shown that adults with PPS struggle to live up to expectations of participating in, e.g., work, childcare, and house-keeping [21], but little is known about how young people meet the challenges of adjusting their activities while also caring for their symptoms.

### The shaping of subjectivity in the face of PPS

Experiences of constraining bodily symptoms force people to reflect on what they can and cannot do and be part of, and on how to live meaningful lives with suffering [22]. As such, living with PPS shapes subjectivity. Within recent anthropological theory on subjectivity, the concept involves a double focus on how people are subject to conditions and at the same time reflect and act on these conditions. Subjectivity thus refers to inner life processes and states of affect, agency, and morality that are shaped through social experiences that take place in specific sociocultural contexts [22]. Hartmut Rosa’s concept *social acceleration* encompasses important aspects of the sociocultural environment that young people live in [23]. Put very shortly, Rosa argues that because of several processes of acceleration, people experience an increased time pressure in their lives. While a technological acceleration has made it possible to produce, transport, and communicate faster, we fill out the time gained from this with more and more tasks and activities and in this way, the pace in our lives has accelerated. The late modern human being thus faces an exploding to-do list with *“things we have to do, things we feel we should do, and things we want to do”* [24, p.82]. Youth researchers have pointed to the significance of social acceleration for experiences of mental distress [24], yet less focus has been given to how they are interwoven with experiences of physical symptoms.

## Methods

The study is based at the Research Clinic for Functional Disorders and Psychosomatics and the Department for Child and Adolescent Psychiatry at Aarhus University Hospital, Central Region Denmark. The authors have different scientific backgrounds (anthropology, psychiatry, psychology, public health, and general practice) and are all experienced researchers within research on symptoms and functional disorders. JHA and MBR have extensive experience in qualitative health research. In line with the research focus on how young people experience and manage their symptoms, we chose a qualitative research design involving interviews and photo-elicitation.

### Recruitment of participants

The first author (JHA) recruited 11 young people through general practitioners, primary care paediatricians, and Facebook based on the following inclusion criteria: (1) age: 15–25 years, (2) have experienced PPS for at least 2 months (ongoing or recurrent) that affect daily activities, and (3) read, speak, and understand Danish. The following exclusion criteria were applied: (1) the symptoms are fully explained by an existing somatic disease or psychiatric disorder; (2) the symptoms have caused long-term absence from work or school (= 100% for 3 months); and (3) known problematic use of drugs or alcohol. These criteria were chosen in order to recruit young people with mild to moderate PPS rather than a chronic condition, as the former are the target group for the *My Symptoms Young* programme our study contributes to. The participants were recruited through two GPs and three private practising paediatricians based in Central Denmark Region and the Region of Southern Denmark and through a public post on Facebook. The GPs and paediatricians gave eligible young patients brief information about the project and asked for permission to provide JHA with the patient’s contact details. JHA subsequently phoned the patients in order to introduce them to the project, ask for their consent to participate, and arrange an interview. The young people who were recruited through Facebook contacted JHA after having seen a public post regarding the project. By recruiting young people in this way, we reached people from different parts of Denmark and obtained a good variation with respect to the young people’s age, types of symptom, and experiences with medical treatment (see Table 1). However, only one male was recruited.

**Table 1:** Participant characteristics

Name*	Gender	Age-range	Symptoms	Duration of symptoms (years)	Education/work
Yasmin	F	15–20	Headache, nausea, dizziness, visual disturbances, tiredness	1	Secondary School
Mette	F	15–20	Pain in back, knees, ankles, feet	2	Secondary School
Sidsel	F	15–20	Stomach ache	2	Secondary School
Sofie	F	17–20	Stomach ache, migraine, back pain, headache	> 5	Secondary School
Anna	F	21–25	Pain in knees, hips, wrists, headache	> 5	Vocational education
Luisa	F	21–25	Headache, stomach ache	3	Work
Carla	F	15–20	Pain in the back, neck, hips and knees	> 5	Upper Secondary School
Nora	F	15–20	Headache	> 5	Secondary School
Olivia	F	15–20	Stomach ache, sleep problems, heart pounding, headache	2	Upper Secondary School
Martin	M	21–25	Headache, back pain, sleep problems	> 5	University
Naja	F	21–25	Pain in the back and neck, sleep problems	1	Vocational education

*These names are not the participants real names

### Interviews

JHA carried out the interviews from April to June 2021. Due to the COVID-19 situation and the geographical distance, 10 interviews were carried out online and only one was done face to face. All interviews were audiotaped and transcribed. The first part of the interviews consisted of a broad exploration of how the participants’ everyday lives were affected by the symptoms; the practices they used to try to manage their symptoms and which challenges they experienced in this regard; which resources they drew on in their approach to their symptoms; who they sought care from; and how they experienced the professional care that they received from their physician (GP or paediatrician).

JHA used photo-elicitation to facilitate the first part of the interviews. Prior to the interviews, JHA instructed the participants to take two photos of a pleasant situation and two photos of a situation, in which they felt bothered by the symptoms. Taking photos was optional and seven participants chose to do so. During the interview, the participants showed the photos to JHA, who asked them to elaborate on what was on the pictures and why they had been taken. After the interview, the photos were sent to JHA by email. Through talking about the photos the participants anchored their symptom experiences and emotions to specific situations including the activities, persons, and artefacts that were part of that situation [25].

The second part of the interview focused on the potentials for redesigning the *My Symptoms* self-help programme to fit young people, but this issue is not covered in this article.

### Analysis

JHA conducted a thematic analysis of the interviews [26]. The photos were not analysed in themselves, rather what was said about them was analysed as part of the overall analysis of the interviews. The thematic analysis involved a systematic process starting with a thorough reading of the whole material. Next, as the first step in organizing the material, interviews were coded using Nvivo. Based on a thorough examination of the codes, possible themes and subthemes were identified. These themes were then further developed in loops of writing and discussions with two of the co-authors (CUR and MBR). While developing the themes, JHA continually returned to the codes and the interviews to check the consistency of the analysis. Through this process, we decided that the central themes in the interviews were how the participants figured out which activities to participate in and how norms of being social and active challenged their efforts at adjusting their activity levels in order to avoid provoking or exacerbating their symptoms. The final analysis thus evolves around these themes, which have been explored by drawing on the concept of subjectivity and theory about social acceleration presented in the introduction section.

## Results

It was characteristic of the young people in our study that they were engaged in many things in their everyday lives. They had school, work, sports, hobbies, and friends that kept them busy, and although the symptoms affected their engagement, everyone was either in school or at work and had part-time activities.

Across the interviews, the challenge of balancing daily activities to avoid provoking or exacerbating symptoms was a dominant theme, and most participants had to adjust their activity level. In the following, we explore the participants’ thoughts on making these adjustments and the challenges they faced.

### Staying as engaged as possible

The participants kept being engaged in many activities in spite of their symptoms. When JHA asked Yasmin, who suffered from persistent headaches, dizziness, visual disturbances, nausea, and tiredness after a concussion, what she did if she woke up with symptoms, she said that she just did what she would normally do (go to school, hang out with friends, play her instrument). She explained:

*I just do what I would normally do because I don’t really know what to do to make it go away (…). I have a headache every day, some days more than others, so I just do what’s normal, but if it is really bad, I go home (…). But there is not much else to do than wait for it to go away.*

Similarly, Sidsel and Sofie said that they just endured their stomach aches, because they had been told by their paediatricians that there was nothing that could be done except taking laxatives to prevent constipation. Thus, when symptoms made it hard to concentrate in school, for example, the participants would usually stick it out. At the same time, the participants did a number of things to alleviate their symptoms. Sofie stressed that she followed all the general advice for healthy eating and exercising, although it had not helped her. Sidsel explained that eating was often accompanied by stomach pain and a need to go to the toilet. Without really being able to explain why, she did not want to have to run to the toilet many times during the school day, so she had developed a strategy of not eating or only eating a little bit in school. Other participants mentioned taking pain killers, using ice packs on hurting joints, blue light glasses for concussion-related headache, stretching, and resting. Most also received treatment from doctors or therapists (e.g., physiotherapists). The participants thus did a number of things to stay as engaged in daily activities as possible, taking the necessary precautions. As we return to below, most participants had also cancelled activities: some had stopped doing sports, others skipped social arrangements or stayed home from school when symptoms were really bad. Yet, we found that the general sentiment was that when symptoms are constant and there is no immediate solution to them, a more general withdrawal from daily activities is not a solution.

### Figuring out which activities to participate in

Instead of withdrawing from many activities, the participants attempted to adjust their participation in activities to not provoke or exacerbate symptoms. The participants’ processes of figuring out which and how many activities to take part in can be described as one of trial and error and careful deliberation. One of the photos taken by Mette depicted her class playing a ball game [*Rundbold*], while she was sitting on the bench. Mette explained that often she could not participate in physical education in school due to her knee pain. The pain waxed and waned during the week, so she had to make decisions on a day-to-day basis. She had learned that ball games, where you run and then stop suddenly, were especially bad for her knees, so often she did not participate in such games. However, she was also sick and tired of either being on the bench or counting the score, so sometimes she decided to participate anyway. Deciding what to do on any given day involved evaluating the severity of the symptoms and was not easy. Sometimes she called her mom to discuss the matter:

*It is darn hard. I also often call my mom [to ask], ‘can I take part in that’? She says, ‘well, I don’t know’. It is so hard to have to be the judge yourself of what you can and what you cannot do because I want to do everything, but after one and a half years, I have learned that I cannot do it all.*

Like Mette, other participants were involved in ongoing assessments of how activities influenced their symptoms, and they discussed the matter with their parents and therapists in the attempt to find patterns. Making these assessments and drawing conclusions from them was difficult. Even if they could identify a connection between an activity and the severity of their symptoms, which many could not, the degree to which an activity would exacerbate the symptoms could differ. For example, Luisa explained that using her computer or watching TV provoked her headache, but it was unpredictable how long she could do so before the headache would set in. In spite of the participants’ attempts to evaluate symptoms and draw connections, the symptoms remained somewhat unpredictable and thus it was not possible to gain control over them.

Deciding which activities to engage in was also difficult because activities often had a social element to them that the participant did not want to miss – they still wanted to do everything, as Mette explains above. She explained how she longed to be part of the ball game because she could see that the players were having fun, which stressed the social aspect of it. Similarly, Yasmin explained how missing a weekend trip with her friends was ‘a bummer’:

*Recently, I was supposed to go with my friends [to a summer house] for a weekend, but I cancelled because that week I had a lot of pain in my neck, nausea, and headache and I couldn’t. Well I really wanted to go, but I couldn’t do it because we wouldn’t be taking it easy (…). It was really a bummer that I couldn’t go because you could see in the stories [on social media] that they were all having such a good time, so you felt a little left out but you knew that it wasn’t because you were left out, it was because you couldn’t come.*

Similar to Yasmin and Mette, other young people said that having to refrain from activities came with a feeling of not being part of the group, of missing out, and of being different.

All the participants tried to take action towards their symptoms and to limit the influence on their lives, now and in the future, by alleviating measures and adjusting their activities. Yet, doing so jeopardized their sense of being part of important social relations. Although they all talked about their friends and nobody seemed socially isolated, an insecurity concerning their place in social networks thus accompanied their attempts to act sensibly with regard to their symptoms.

### Adjusting activities when being *social* is the norm

In addition to their own concerns about standing outside the group, the participants’ accounts also painted a picture of a norm of being social, which they had to go against, when trying to opt out of activities with their friends due to their symptoms. The responses from understanding friends reflect that being social is expected of young people. For example, Yasmin and Nora explained how their friends – in the best of intentions – would ask them if they were sure that they did not want to hang out, and Yasmin told how her friends had offered painkillers and assured her of the possibility to rest during a trip because ‘*sometimes it is more fun when you are more people together’*. For Nora, the norm of being social was not only a challenge due to her symptoms, it also challenged her sense of self. After having seen a psychologist and read a book on the subject, she realized that she was an introvert person. She also discovered that some of her headaches started when she had many plans or was around many people, and she therefore tried to take more time to be herself in the hope that some of her headaches would disappear. Yet, she found it difficult to tell her friends that she had to be on her own sometimes because she was afraid that they might take it personally or be disappointed.

Although most participants said that their friends accepted it when they were unable to participate in activities because of their symptoms, they also assumed that their friends did not really understand their symptoms and how it affected them, and that some might be sceptical towards the duration of the symptoms. Luisa, who had to withdraw from almost everything for a period following a severe concussion, explained:

*Quickly, you feel that you have to come up with an excuse, sort of like it is not enough that I’m in pain again. (….) And you feel people are thinking, how far can she push it, she can’t be in that much pain.*

Participants also said that the lack of clear connections between symptoms and activities made it hard to explain how they chose between activities. As Mette speculated: *I can imagine that it looks really weird that you can go for a run but you cannot participate in M-ball.* Some also mentioned that the lack of visible signs contributed to the difficulty of explaining the symptoms. Although few reported being confronted with other people’s scepticism towards their symptoms, it was evident that the lack of an adequate explanation made many participants uncertain about the social legitimacy of their symptoms and of their prioritization of activities.

### Adjusting activities when being *active* is the norm

Some participants engaged in activities although it made their symptoms worse. Anna suffered from widespread pain. She had been told numerous times by professionals that she should cut down on her plans; however, she had not been able to do so:

*Yesterday evening I was actually watching television, which I don’t do very often because I don’t have time for it at all, and that is probably one of the things that I struggle most with: that all the smart people around me, psychologists and physiotherapists and all the doctors, tell me: ‘Anna, you have to slow down, you should not have so many plans and you have to rest’. I do not know why I have so many plans; I feel that I am trying to say no and to coordinate things, but it is just characteristic of my life that I am constantly busy and constantly on the move.*

Although this quote illustrates her experience of how her many plans are almost beyond her control, Anna’s account was ambiguous. She acknowledged that she was close to having stress and that this might influence her pain. At the same time, she could not figure out which activities to drop, and she thrived with being busy and did not want her pain to control how she lived her life. Thus, she was torn between recognizing that her preferred way of living contributed to her symptoms, and fighting the symptoms to maintain this way of living. Olivia’s account had parallels with Anna’s. Oliva struggled with stomach ache, difficulty sleeping, heart pounding, and headaches, which she interpreted as being stress related. She too found it very difficult to prioritize between her daily activities. Furthermore, Olivia explained that her stomach ache was related to relaxing; if she did something relaxing such as watching TV, she would get a stomach ache from thinking about all the more productive things she should be doing. She had previously seen a psychologist, but it had not worked out, and now she just did not have time to deal with it. Similar to Anna, she thus found that it was easier to just keep going than to recognize the symptoms and figure out how to handle them.

It was a general feature of the participants’ stories that their symptoms were an unwanted interference in their busy lives. While Olivia and Anna made direct connections between stress and their symptoms, others talked about feeling stressed or pressured but did not relate it to their bodily symptoms, and some said that their sleep difficulties (e.g., finding it hard to fall asleep) was a stressor because they would lie awake thinking about how they should manage all their plans the next day when lacking sleep.

There were a few examples of participants skipping part-time activities they no longer had time for, and in our talks about their everyday lives, some did mention relaxing in the afternoon or evening. For example, one of Sidsel’s pictures of a pleasant situation depicted her bed, and she explained that she enjoyed sitting in her bed watching Netflix. Still, the general impression was that the normal thing was to have many things going in one’s life, and in some cases the many plans almost seemed to live a life of their own, leaving the participants unable to prioritize and find time to relax. Even those who recognized the link between their symptoms and their many activities and stress felt unable to do something about it.

## Discussion

This study shows how young people with PPS attempt to manage their symptoms while staying engaged in their everyday activities and it illuminates the social concerns and norms involved in these attempts. The young people’s subjectivity is mediated by their symptoms, affecting their perceptions of what they can and cannot do, thus making them reflect on their attachment to social networks and the social legitimacy of their symptoms.

The participants attempted to take responsibility for their symptoms and their lives in general by trying different ways of relieving their symptoms and find patterns between specific activities and their symptoms in order to adjust their activities instead of withdrawing entirely from their usual social engagements. As such they act as ‘good’ patients [27]. However, recognizing patterns between activities and symptom severity was difficult, and when trying to adjust their activities, their sense of belonging to social networks was jeopardized, and they faced imperative norms of being social and active. As PPS shape the participants’ sense of how to act in relation to their bodies and social relationships, we argue for looking at their subject formations and symptom experiences as a biosocial process [28].

All participants had to opt out of activities from time to time because of their symptoms. In doing so they found it challenging to explain their symptoms to their friends. While only few experienced that their symptoms were disputed by others, they assumed that their friends did not really understand the symptoms and might be sceptical towards their duration. The issue of how to understand and explain PPS and the accompanying issue of social legitimization have been shown in other studies of young people [14, 18] and adults with PPS [29, 30]. Historically, the lack of clinical or para-clinical findings in patients suffering from PPS has given rise to speculations among lay people and health professionals that the symptoms are not real in a biological sense but stem from either psychological distress or are simply made up [29]. In consequence, patients’ experiences of such symptoms may be delegitimized by others. In our study, the lack of clear associations between activities and symptom severity and the lack of a diagnosis and visible signs of their symptoms made the participants uncertain of the social legitimization of their symptoms, and thereby of their decisions concerning which activities to participate in.

The participants were typically engaged in several activities in their everyday lives, and prioritizing between them in order to prevent symptom exacerbation or stress was difficult. Although they were engaged in activities out of interest and because they enjoyed it, in some cases it seemed that the participants had almost become subject to their own plans. Their depiction of their everyday lives illustrates the aspect of Rosa’s concept of social acceleration that concerns how human beings have increased the number of daily activities and therefore experience an increasing time pressure [23]. In a large mixed-methods study on experiences of poor mental well-being among young Danes, Katznelson, Pless and Görlich [24] build on Rosa’s concept of social acceleration: they identify three ways young people experience acceleration, tempo, and time pressure, namely as *everyday acceleration*, *institutional acceleration*, and *life biographical acceleration*, and they argue that these types of acceleration contribute to the high level of mental distress among young people. *Everyday acceleration* refers to how young people’s everyday lives are marked by a very high pace where activities accumulate while they lack the tools they need to prioritize what is most important [24]. This insight is thus directly paralleled by our argument on the norm of being active. *Institutionalized acceleration* refers to how the educational system, especially in upper secondary school and university, is marked by increased expectations towards finishing fast while also performing well, and *life biographical acceleration* entails that young people feel a pressure to make the right choices for their future fast (especially in relation to education and work life), and that they feel an individual responsibility for succeeding with very limited room for making mistakes. In our study, experiences that can be related to institutionalized and life biographical acceleration were evident in the participants’ determination to stay in school and meet their educational responsibilities. We thus argue that the different forms of acceleration are important to bear in mind when trying to understand why it can be difficult for young people with PPS to adjust activities even when they feel pressured and stressed and attribute this to their physical symptoms.

### Strengths and limitations

The interviews for this study were carried out by a researcher (JHA) who has extensive experience in doing qualitative research with young people, and the data analysis was conducted in dialogue with a cross-disciplinary research group with extensive experience in symptom research. The semi-structured interview format allowed participants to talk freely about what mattered to them in relation to their symptoms. JHA used photo-elicitation to make it possible for the participants to capture the specific daily situations in which they were or were not bothered by their symptoms, and it helped them provide details about these situations in the interviews. However, those who had not taken photos were also good at connecting their symptom experiences to specific situations.

Given the double focus on everyday experiences of PPS and the potential of developing *My Symptoms Young*, a lot had to be covered in the interviews. Follow-up interviews might have provided a deeper insight into how the participants experienced and handled their symptoms over time and the challenges they faced in this connection.

The participants were a very heterogonous group with respect to the number, severity, and duration of their symptoms as well has how much medical treatment, they had received. They were different with respect to how bothered they were by their symptoms in their daily lives. This diversity is a strength in that it corresponds to the diversity in patients with PPS seen in primary care. However, it is a limitation of the study that we were only able to recruit one male participant. Although there were no apparent gender differences in the experience and handling of PPS among the study participants, a greater number of male participants might have made possible differences apparent. It is therefore relevant for future research on PPS to engage with the gender perspective to explore possible gendered ways of managing symptoms.

## Conclusion

This study shows that having PPS affects young people’s sense of how to act in relation to their bodies and social relationships, and we argue for looking at their subject formation and symptom experiences as a biosocial process. The participants attempted to take responsibility for their symptoms by using different means of alleviation and adjusting their activities rather than completely withdrawing from their everyday engagements. In trying to adjust activities, they experienced difficulties finding patterns between symptom severity and activities, and they came to reflect on their attachment to social networks and were challenged by norms of being social and active. They also found it difficult to explain their symptoms to others and were uncertain about the social legitimacy of their symptoms and prioritization of activities.

Our study shows that young people’s experiences of PPS and their attempts at adjusting their activities to care for their symptoms are affected by larger societal trends of social acceleration. Still implications for clinical practice can be drawn from the analysis. Health care professionals working with young people with PPS should be able to provide knowledge on how to understand symptoms and tools for identifying patterns between activities and symptom severity. Preferably health care professionals should also help young people with PPS with practical advice on how to prioritize between activities, say no to activities that requires too much energy, and find time for self-care.

## Data Availability

The dataset used and analysed during the current study is available from the corresponding author on reasonable request.

## Abbreviations

PPS: Persistent Physical Symptoms
GP: General Practitioner
Yo-eASY: Young ehealth and GP Assisted programme for unspecific SYmptoms

## References

[1] Rasmussen M, Kirkegaard L, Rosenwein SV, Holstein BE, Damsgaard MT, Due P, Skolebørnsundersøgelsen. 2018, Helbred, trivsel og sundhedsadfærd blandt 11-, 13- og 15-årige skoleelever i Danmark. [The school children study 2018. Health, well-being and health behaviour among 11-, 13- and 15-year-old school children in Denmark]. Statens Institut for Folkesundhed, SDU; 2019.

[2] Ottosen MH, Andreasen AG, Dahl KM, Hestbæk A, Lausten M, Rayce SB (2018). Børn og unge i Danmark velfærd og trivsel 2018 [Children and youth in Denmark, welfare and well-being 2018].

[3] Ottová-Jordan V, Smith ORF, Gobina I, Mazur J, Augustine L, Cavallo F (2015). Trends in multiple recurrent health complaints in 15-year-olds in 35 countries in Europe, North America and Israel from 1994 to 2010. Eur J Public Health.

[4] Sundhedsstyrelsen. Danskernes sundhed - den nationale sundhedsprofil 2022. [The health of the Danish population - the national health profile 2022] Sundhedsstyrelsen; 2022.

[5] Rask MT, Jakobsen PR, Clemensen J, Rosendal M, Frostholm L (2021). Development of an eHealth programme for self-management of persistent physical symptoms: a qualitative study on user needs in general practice. BMC Fam Prac.

[6] Hoftun GB, Romundstad PR, Zwart J-A, Rygg M (2011). Chronic idiopathic pain in adolescence – high prevalence and disability: the young HUNT study 2008. PAIN®.

[7] Vila M, Kramer T, Obiols JE, Garralda ME (2012). Abdominal pain in british young people: Associations, impairment and health care use. J Psychosom Res.

[8] Steinhausen H-C, Winkler Metzke C (2007). Continuity of functional-somatic symptoms from late childhood to young adulthood in a community sample. J Child Psychol Psychiatry.

[9] Hetlevik Ø, Meland E, Hufthammer KO, Breidablik HJ, Jahanlu D, Vie TL (2020). Self-rated health in adolescence as a predictor of ‘multi-illness’ in early adulthood: a prospective registry-based norwegian HUNT study. SSM - Population Health.

[10] Garralda ME, Bailey D (1987). Psychosomatic aspects of children’s consultations in primary care. Eur Arch Psychiatr Neurol Sci.

[11] Starfield B, Gross E, Wood M, Pantell R, Allen C, Gordon IB (1980). Psychosocial and psychosomatic diagnoses in primary care of children. Pediatr (Evanston).

[12] Klastrup L, Rask M, Rosendal M, Christensen K, Rask C (2022). Functional somatic symptoms in youths in general practice, a cross-sectional study on prevalence, clinical management and experienced burden. J of Psychosom Res.

[13] Sitnikova K, Leone SS, van Marwijk HWJ, Twisk J, van der Horst HE, van der Wouden JC (2019). Effectiveness of a cognitive behavioural intervention for patients with undifferentiated somatoform disorder: results from the CIPRUS cluster randomized controlled trial in primary care. J Psychosom Res.

[14] Buchbinder M (2015). All in your head: making sense of pediatric pain.

[15] Kvamme MF, Wang CEA, Waage T, Risør MB (2020). Fixing my life’: young people’s everyday efforts towards recovery from persistent bodily complaints. Anthropol Med.

[16] Østbye SV, Kvamme MF, Wang CEA, Haavind H, Waage T, Risør MB (2020). Not a film about my slackness’: making sense of medically unexplained illness in youth using collaborative visual methods. Health.

[17] Hulgaard DR, Rask CU, Risor MB, Dehlholm G (2020). Illness perceptions of youths with functional disorders and their parents: an interpretative phenomenological analysis study. Clin Child Psychol Psychiatry.

[18] Moulin V, Akre C, Rodondi P-Y, Ambresin A-E, Suris J-C (2015). A qualitative study of adolescents with medically unexplained symptoms and their parents. Part 1: experiences and impact on daily life. J Adolesc.

[19] Moulin V, Akre C, Rodondi PY, Ambresin AE, Suris JC (2015). A qualitative study of adolescents with medically unexplained symptoms and their parents. Part 2: how is healthcare perceived?. J Adolesc.

[20] Laursen SS, Dehlholm-Lambertsen B, Stenager E, Johannessen H (2019). Morality in clinical space: treatment of youngsters with functional somatic symptoms in a western clinical context. Anthropol Med.

[21] Risor MB, Fainzang S, Hem HE, Risør MB (2010). Healing and recovery as a social process among patients with medically unexplained symptoms (MUS). The taste for knowledge: Medical Anthropology facing medical realities.

[22] Biehl J, Good BJ, Kleinman A, Subjectivity (2007). Ethnographic Investigations.

[23] Rosa H (2013). Social Acceleration.

[24] Katznelson N, Pless M, Görlich A (2022). Mistrivsel i lyset af tempo, præstation og psykologisering: om ny udsathed i ungdomslivet. [Poor well-being in the light of pace, performance and psychologization] 1. Udgave.

[25] Guillemin M, Drew S (2010). Questions of process in participant-generated visual methodologies. Visual Stud.

[26] Braun V, Clarke V (2006). Using thematic analysis in psychology. Qual Res Psychol.

[27] Risør MB, Lillevoll K (2021). Caught up in Care: crafting Moral subjects of chronic fatigue. Med Anthropol.

[28] Seeberg J, Roepstorff A, Meinert L, Seeberg J, Roepstorff A, Meinert L (2020). Introduction. Biosocial Worlds: Anthropology of health environments beyond determinism.

[29] Ware NC (1992). Suffering and the Social Construction of illness: the delegitimation of illness experience in chronic fatigue syndrome. Med Anthropol Q.

[30] Risør MB (2009). Illness explanations among patients with medically unexplained symptoms: different idioms for different contexts. Health.

[31] The Act on Research Ethics Review of Health Research Research Projects and Health Data Research Projects LBK nr. 1338 af 01/09/2020 Bekendtgørelse af lov om videnskabsetisk behandling af sundhedsvidenskabelige forskningsprojekter og sundhedsdatavidenskabelige forskningsprojekter (retsinformation.dk).

